# Evaluation of the accuracy of a mandatory indication field for outpatient antibiotic prescriptions

**DOI:** 10.1017/ash.2022.368

**Published:** 2023-02-27

**Authors:** Johanna Papanikolla, Adam Bursua, Jonathan Radosta, Scott A. Borgetti, Susan Bleasdale, Elizabeth Van Dril, John Shilka, Alan E. Gross

**Affiliations:** 1 Department of Pharmacy Practice, University of Illinois at Chicago College of Pharmacy, Chicago, Illinois; 2 Hospital Pharmacy Services, University of Illinois Hospital and Health Sciences System, Chicago, Illinois; 3 Ambulatory Services Administration, University of Illinois at Chicago, Chicago, Illinois; 4 Division of Infectious Diseases, Department of Internal Medicine, University of Illinois at Chicago, Chicago, Illinois

Although antibiotic stewardship programs (ASPs) have an established role in the acute-care setting, ASPs remain nascent in outpatient settings.^1^ Yet, 80%–90% of antibiotic use in humans occurs in outpatient settings, and the Centers for Disease Control and Prevention (CDC) estimates that nearly 50% of this prescribing is inappropriate.^2–4^ The Joint Commission recently introduced ASP requirements for accredited ambulatory care organizations.^5^

It is vital for institutions to assess their general prescribing practices to set a foundation for future stewardship efforts. The use of antibiotic indication fields within computerized prescriber order entry (CPOE) is advocated for by the CDC to facilitate benchmarking and communication.^1^ Several institutions have investigated the accuracy of prescriber-selected indications for inpatient antibiotic orders, but none have assessed the accuracy of outpatient antibiotic indication fields.^6–9^ The objective of this study was to validate the accuracy of the indication field upon antibiotic prescribing in the ambulatory care setting so this field may be used to for benchmarking to facilitate future stewardship interventions.

## Methods

This retrospective quality assurance project was conducted within an existing ASP to validate the accuracy of a mandatory antibiotic indication field when prescribing outpatient antibiotics. This study was conducted at an academic medical center with multiple ambulatory care clinics, urgent care clinics and an emergency department (ED). The primary end point was the proportion of indication fields that accurately reflected the reason the antibiotic was prescribed. We utilized an electronic health record (EHR)–specific reporting tool, Epic Reporting Workbench (Epic Systems, Verona, WI), to identify patients aged ≥18 years who received an outpatient oral antibiotic prescription between September 1, 2021, and October 22, 2022. Investigators extracted the antibiotic order date, prescriber name, drug name, dose, directions, duration, and selected indication from the medical record. The mandatory indication field includes checkboxes for common outpatient infectious syndromes listed in Table 1, as well as an “other” free-text option. Patients were only included once.

**Table 1.** Indication Frequency for Antibiotics Prescribed in the Outpatient Setting

Selected Indications	Prescriptions (N = 350), No. (%)
Skin/soft-tissue infection	85 (24.3)
Urinary tract infection	73 (20.9)
Genital tract infection	72 (20.6)
Other	38 (10.8)
Prophylaxis or chronic suppression	24 (6.8)
Sinusitis/otitis, acute bacterial	17 (4.8)
Pharyngitis, bacterial	16 (4.6)
Gastrointestinal	13 (3.7)
*Helicobacter pylori*	5 (1.4)
Bone/joint infection	3 (0.9)
Pneumonia	2 (0.6)
COPD exacerbation	1 (0.3)
Surgical-site infection	1 (0.3)
Shingles/herpes	0

Note. COPD, chronic obstructive pulmonary disease.

Corresponding chart notes were reviewed by an investigator (J.P.) for the outpatient encounter associated with the prescription. If the infection documented in the chart note from the ambulatory visit was consistent with the indication selected upon ordering the antibiotic, then the use of the indication field documentation was considered accurate. In addition, suboptimal use was characterized and defined as the selection of the “other” indication field when there was a more appropriate default option available but entered free text matched the chart documentation. Descriptive statistics were used. This study was approved by the University of Illinois at Chicago Institutional Review Board.

## Results

Among 4,323 outpatient antibiotic prescriptions that met the inclusion criteria, we selected 350 for validation through random sampling. All prescriptions had a corresponding chart note that clearly identified the antibiotic indication. Table 1 presents the frequency of indications in the sample. Of the 350 prescriptions, 62 (17.7%) were prescribed during an emergency department (ED) encounter, with the remaining prescribed in outpatient clinics. The indication field for 324 antibiotic prescriptions (92.5%) was accurate and consistent with the chart documented infection. Of the 26 discrepancies, 16 (4.6%) were inaccurate and 10 (2.9%) were defined as suboptimal. The most common inaccuracy occurred with mismatches between UTIs and genital tract infections (8 of 16). Nineteen discrepancies (73%) pertained to antibiotics prescribed in outpatient clinics, with the remainder from the ED.

## Discussion

Our study is the first to evaluate the accuracy of a required indication field when prescribing antibiotics in the outpatient setting. Overall, 92.5% of selected indications were accurate and matched the infectious syndrome on chart documentation. Accuracy was similarly high among the ED and other outpatient settings, and these indication data can serve as a foundation for benchmarking and systematic stewardship interventions.

Through the course of this review, we identified ways to optimize the indication field listing. Half of the indication inaccuracies were due to prescribers selecting “genital tract infection” instead of “urinary tract infection” for patients with UTIs. Therefore, we have eliminated “genital tract infection” and replaced it with “sexually transmitted infection” to further direct prescribers to the appropriate choice. To provide additional actionable data, we also changed “urinary tract infection” to “UTI, pyelonephritis” and “UTI, cystitis,” and we split “otitis/sinusitis” into 2 unique headings. Furthermore, because the “surgical site infection” indication was selected for only 1 patient and because these infections can be captured by the “skin and soft-tissue infection” indication, we eliminated the former indication.

Prior studies have evaluated the accuracy of the indication field for inpatient antibiotic orders. One of the earliest was conducted at a large academic medical center and evaluated indications entered via CPOE.^6^ These researchers reported that 43 (86%) of the 50 randomized CPOE indications were accurate, with most inaccurate designations being “other.” With the addition of an indication to account for the selection of “other,” it was estimated that accuracy would have improved to 92%. This validation study allowed for additional benchmarking and assessment institutional guideline adherence.

Heil et al^7^ assessed the accuracy of prescriber-selected indications in a pediatric ICU and adult medicine step-down unit. This analysis evaluated the frequency at which antibiotic indications chosen by the prescriber matched the true indications 48–72 hours after the initial order. The study reported a 90% concordance between the provider selected indication and independent review by infectious disease physicians. Similar to our study, one of the highest rates of discordance was seen with genitourinary and urinary tract infections, but this finding was not further elaborated.

In another inpatient medical center, Timmons et al^8^ reported a method for monitoring prescribing patterns by generating indication lists for selected broad-spectrum antibiotics. Indication matching was determined by ensuring the chart-documented diagnosis mirrored the indication in the order. The matching rate was 74%–80%. Notably, prescribers chose “other” for 41% of orders, but in most cases the true indication was not available on the prepopulated indication list. Timmons et al attempted to characterize clinical appropriateness of prescribing as well, and they reported that 13.6% of antibiotic orders were inappropriate. This study sheds light on the scope of potential opportunities to assess clinical appropriateness with validated indication fields.

A recent study by van den Broek et al^9^ focused on respiratory tract infection and UTIs in 3 hospitals in the Netherlands. Similar to our study, prescription indications and chart notes were manually screened, and accuracy ranged from 78.2% to 96.7%.

Through our EHR, we can query specific indication fields used and key elements of the prescription including dose, duration, prescriber, clinic, and other information. These data, when combined with our now-validated indication fields can facilitate benchmarking of outpatient antibiotic use and can serve as a foundation from which to investigate opportunities to improve care. Stratifying these data by prescriber and/or department can shed light on prescribing practices amenable to systematic intervention or provider comparisons. Some system-oriented solutions may include generating indication-based order sets, review and modification of specialty-specific preference lists, modification of order defaults, and implementation of clinical decision support. More targeted antibiotic stewardship interventions may include educational efforts to address inappropriate prescribing patterns for specific prescribers or indications.

Generalizability across institutions is a limitation of this single-center study; additional data are needed from other outpatient centers. Additionally, appropriate use of the indication fields may change over time, which may warrant routine monitoring of this tool. Notably, not all outpatient antibiotic orders have an associated note. Although we did not encounter this within our study, scenarios in which providers enter orders without EHR documentation may occur.

In this retrospective quality assurance project, outpatient prescribers appropriately utilized the mandatory indication function in the CPOE. This project, along with the aforementioned studies, support the importance of a required indication field because it can inform quality care. The indication field offers a method to standardize and support reliable and efficient assessments of antibiotic use. This method provides institutions the opportunity to identify inappropriate antibiotic use and to prompt changes in prescribing practice. Other outpatient centers are encouraged to implement mandatory indication fields for antibiotic orders.
